# Non-Functioning Adrenocortical Carcinoma Presenting as Retroperitoneal Hemorrhage With Early Metastasis

**DOI:** 10.7759/cureus.31665

**Published:** 2022-11-19

**Authors:** Zabih Ullah Khan, Ghazal G Alsisi, Amer Q Aldouri, Fahad W Ahmed, Mohamed Khalid Mohiuddin, Ghaida G Alsisi

**Affiliations:** 1 General and Oncology Surgery, King Faisal Specialist Hospital and Research Centre, Madinah Al Munawwarah, SAU; 2 Medicine, Taibah University, Madinah Al Munawwarah, SAU; 3 Hepatobiliary Surgery, King Faisal Specialist Hospital and Research Centre, Madinah Al Munawwarah, SAU; 4 Diabetes and Endocrinology, University Hospitals Sussex National Health Service (NHS) Foundation, Brighton, GBR; 5 Colorectal Surgery, King Faisal Specialist Hospital and Research Centre, Madina Al Munawwarah, SAU

**Keywords:** early recurrence, surgery, retroperitoneal haemorrhage, spontaneous rupture, non functioning adrenocortical cancer

## Abstract

Adrenocortical cancer is a rare neoplasm with varied clinical presentation and overall poor outcome. This should be managed with timely intervention at highly specialized centers. Our aim is to report this rare case presentation of large non-functional adrenocortical cancer, complicated by spontaneous rupture while awaiting workup leading to life-threatening hemorrhage. Despite successful emergency radical surgical management and achieving negative margins, the patient developed early recurrence as intra-abdominal metastasis within two months. This can likely be attributed to the aggressive nature of the tumor as indicated by the high Ki-67 index or spillage of the tumor cells following spontaneous rupture. We recommend managing these non-functioning adrenocortical cancers as early as possible at highly specialized centers with reference to published standard guidelines.

## Introduction

Adrenocortical cancer is an aggressive rare neoplasm affecting about 0.7 to 2 million adults per year [1,2]. Although peak incidence is seen at 40-60 years, it can affect any age group. It accounts for 0.02% of all malignant tumors [1-3]. There is a slight female predominance. Functional tumors account for 50 to 60% of cases and present with symptoms of clinical hormone excess whereas non-functional tumors may present with nonspecific symptoms like abdominal discomfort, fullness, or back pain. the percentage of incidentally detected adrenocortical cancers is increasing [4]. Only a few of these tumors present with complications like spontaneous rupture, causing hemorrhage [5]. A tumor size greater than 10 cm is a risk factor for spontaneous rupture [6]. Complete surgical resection is the mainstay of management. The prognosis of the tumors is heterogeneous which is also based on multiple independent factors like proliferative activity, tumor grade, emergency presentation, radical surgery, and disease stage. Five-year survival for these tumors is 60-80% when confined to the adrenal gland, 35-50% for local advanced, and 0-28% in case of metastatic disease [7]. 

Here we present a rare case of spontaneous rupture of non-functional adrenal cortical cancer in a young female presenting with early metastases.

## Case presentation

A 37-year-old otherwise healthy female was referred to our tertiary care center from another hospital for further evaluation and management of a large right-sided adrenal tumor detected on a CT scan upon workup of vague abdominal pain. While waiting for her specialized endocrine clinic appointment, she developed acute onset of severe abdominal pain with palpitations and anxiety. Therefore, she was rushed to the emergency department, where she was found to be in severe distress with acute abdominal pain. The patient reported two-month history of dull persistent right flank pain (score of 5/10) associated with intermittent low-grade fever and unintentional weight loss. The pain got worsened over 6-8 hours, generalized with increased severity and sharp nature. The patient denied any symptoms suggestive of functional adrenal tumors like phaeochromocytoma, crushing's or hyperaldosteronism. No significant past medical or surgical history and no family history of any malignancies were reported. She was an active housewife with three children.

She was alert and conscious with blood pressure: 100/80 mm Hg, pulse rate: 128/min, respiratory rate: 24/min, temperature: 36.8 and SpO2: 98% on room air. The patient was administered analgesia and resuscitated with crystalloids. Her review of past medical records from referring center showed normal blood and urine work-up including adrenal hormones (Table 1 shows the blood workup results with hormone levels).

**Table 1:** Preoperative work-up obtained at the referring center The results show adrenal hormonal levels within the normal range; 24-hour urine metanephrine was done outside and was reported as Normal.

Investigation	Result	Range
Cortisol	5.87 ug/dl	AM: 6.02-18.4 PM:2.68-10.5 ug/dl
Aldosterone	6.73 ng/dl	2.21-35.3 ng/dl
Plasma Renin	25.37 u IU/ml	4.4-46.1 u IU/ml
Aldosterone/Renin ratio	0.265	0.1-3.7

**Figure 1:**
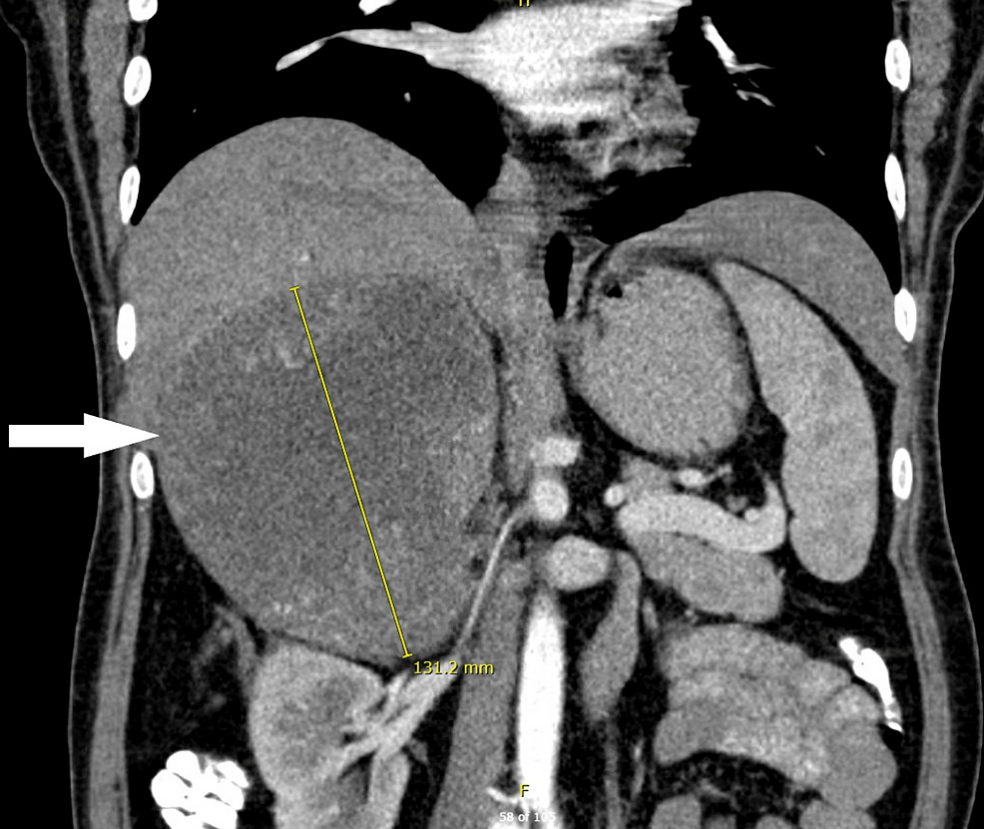
CT scan abdomen (coronal view) from the referring center The image shows an intact, well-defined large adrenal mass (white arrow) with central hypo density and heterogenous contrast enhancement, it shows a compression effect on the right kidney and liver. There are no obvious intra-abdominal metastases.

**Figure 2:**
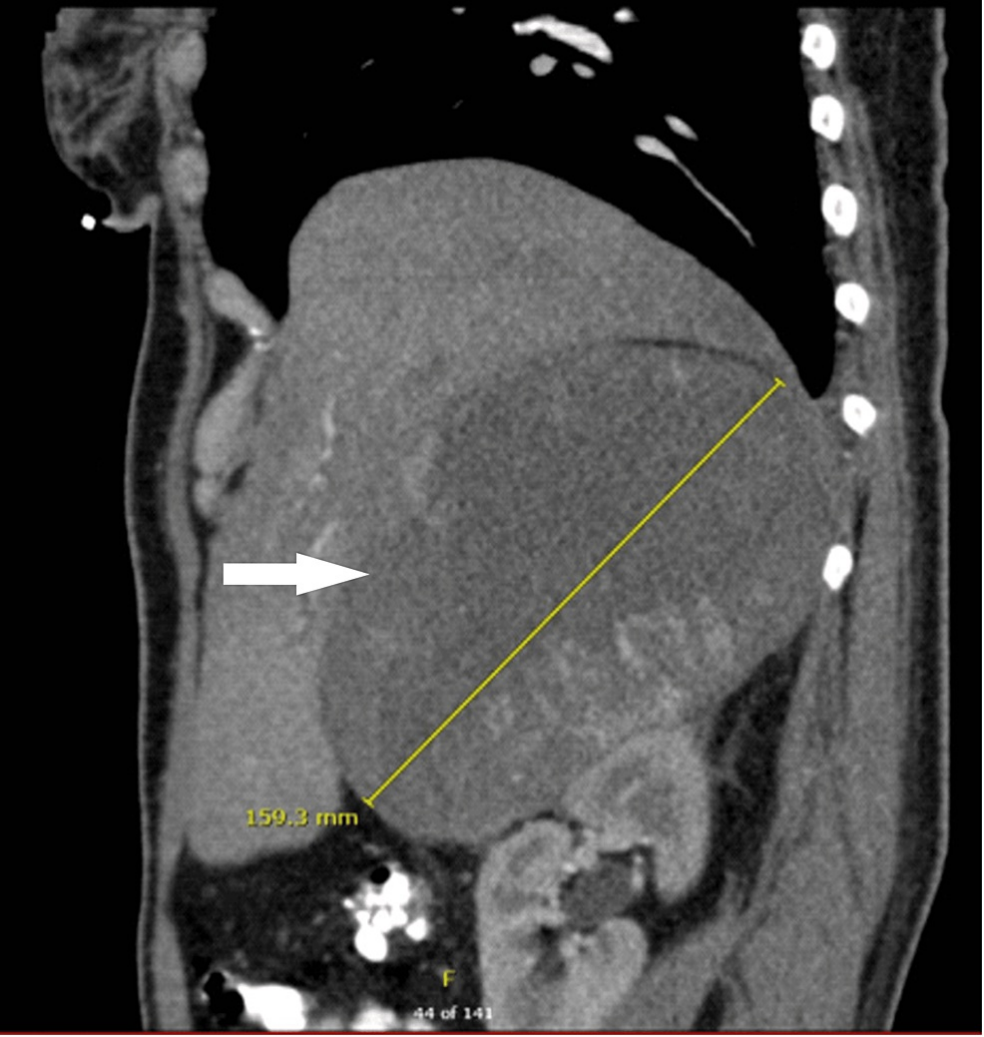
CT scan abdomen (sagittal view) from the referring center The image is showing the adrenal tumor (white arrow) with a maximum dimension of 15 cm.

Her imaging review at another hospital showed a 12.5x11.6x15 cm suspicious adrenal tumor with heterogeneous enhancement and central necrosis with mass effect on the liver (Figures 1, 2).

Keeping in view the background history of adrenal mass, acute onset severe abdominal pain, and early signs of shock, a contrast-enhanced computed tomography (CECT) was obtained at the emergency department to rule out tumor-related complications. 

**Figure 3:**
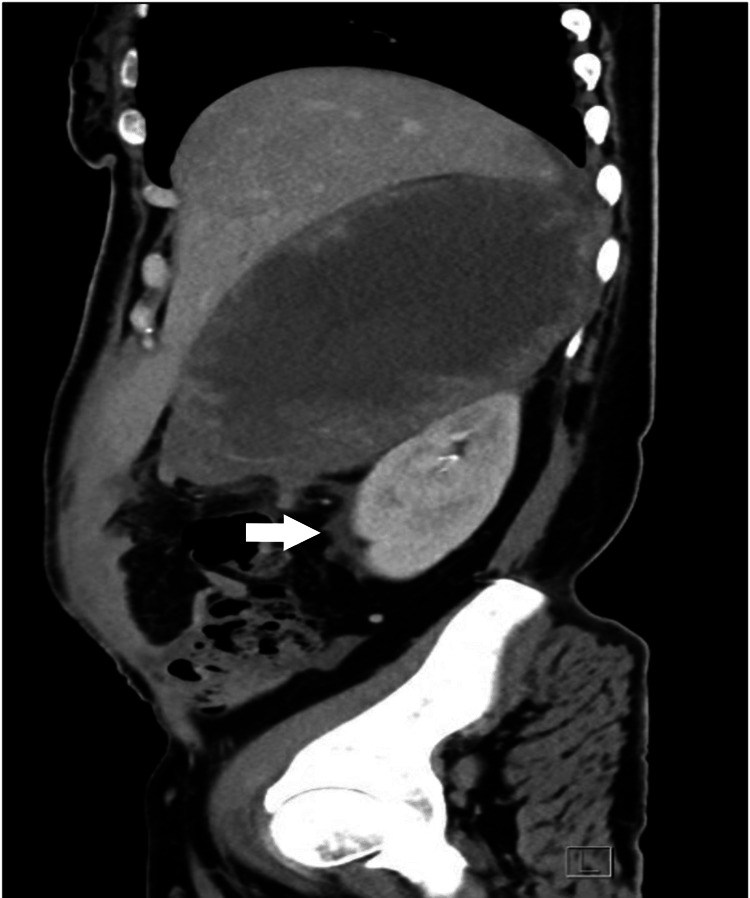
CT scan abdomen (sagittal view) obtained at the emergency department showing tumor rupture with retroperitoneal hemorrhage (white arrow)

**Figure 4:**
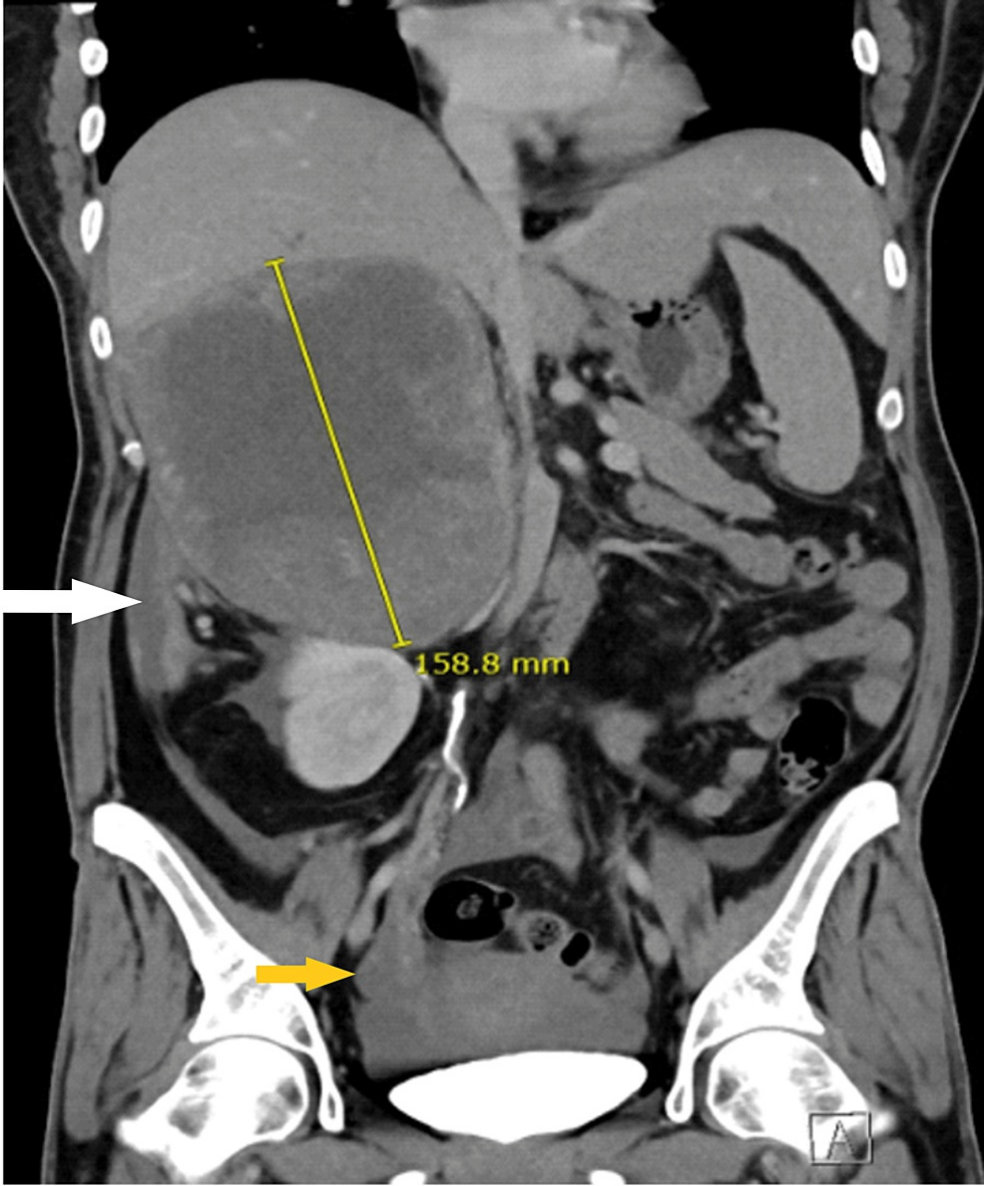
CT scan abdomen (coronal view) obtained at the emergency department The image is showing retro-peritoneal (white arrow) and intraperitoneal (yellow arrow) hemorrhage from the tumor.

**Figure 5:**
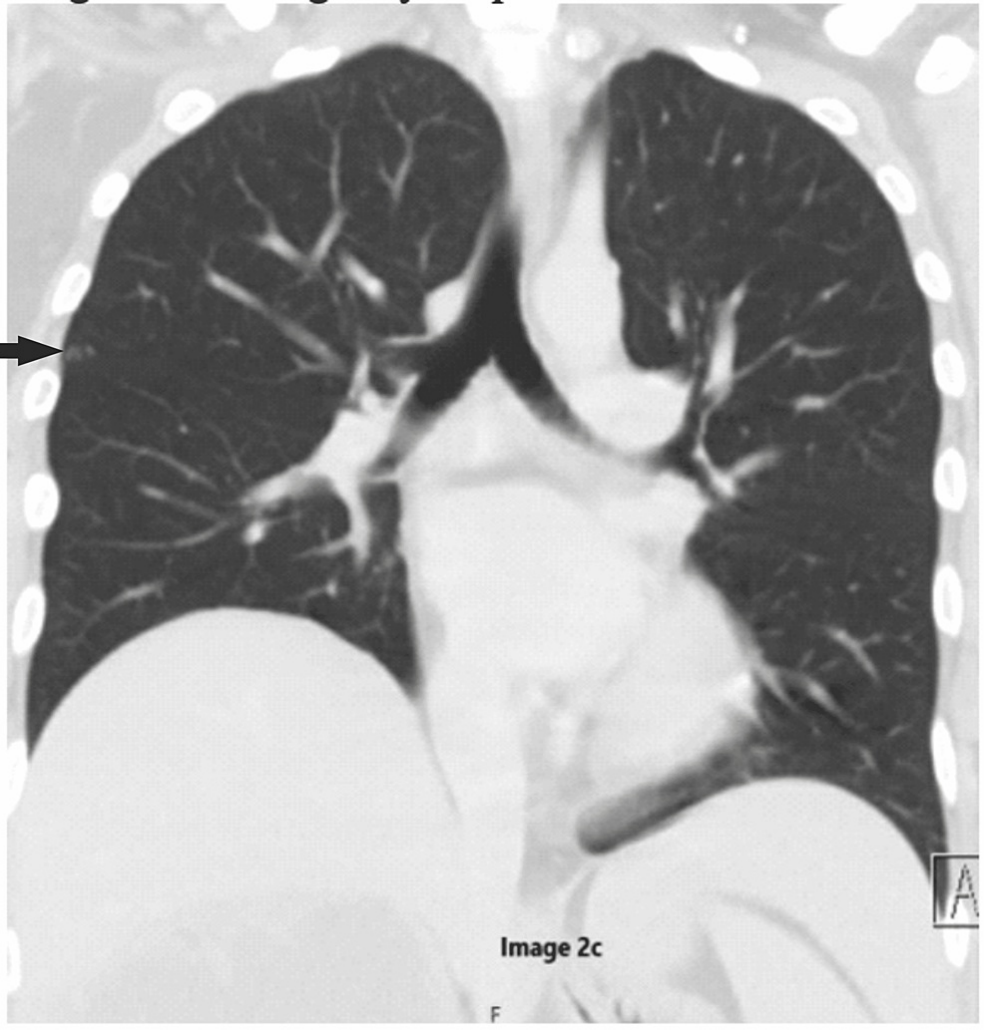
Initial CT scan chest (sagittal view) The image is showing a tiny suspicious right lung lesion (black arrow).

The scan showed a huge 12.5x13.7x18 cm right adrenal tumor with evidence of rupture, retro-peritoneal and mild to moderate intra-peritoneal hemorrhage, heterogenicity, central necrosis and suspected invasion of the liver parenchyma, suggestive of malignancy. No distant metastases were found. (Figures 3-5).

With suspected hemorrhage of this adrenal mass, the patient was transferred to the intensive care unit with planned emergency angiogram +/- angioembolization of the feeding vessel.

**Figure 6:**
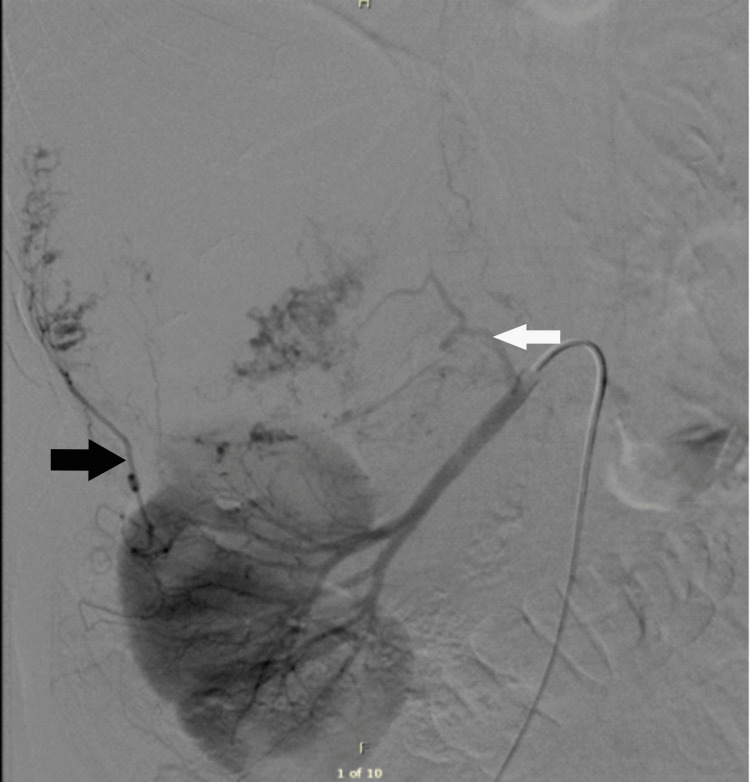
Emergency angiogram The image is showing suprarenal mass and feeding vessels: adrenal artery from right renal artery (white arrow), direct feeding branch from superior renal vessels (black arrow). There was no active bleeding at the time of the angiogram.

The angiogram revealed a large right suprarenal mass with neovascularity in its central portion and lateral portion. The central portion was obtaining the blood supply from the right adrenal artery which is arising from the right renal artery. The lateral portion is obtaining the blood supply from a right renal capsular branch. Due to the lack of a specialized catheter for embolization, the angio procedure could not be completed; moreover, there was a lack of clear visualization of active bleeding on the angiogram (Figure 6). Hence a decision was made to treat the patient conservatively in the intensive care unit for closed observation with a backup plan for emergency surgical intervention in case the patient deteriorates.

However, the patient’s condition deteriorated in the intensive care unit with a drop in hemoglobin of 4 grams (Hb 11 to 7g/dl) and she became hemodynamically unstable with tachycardia - 140/minute and BP 90/70 mm of Hg. The patient was transferred for emergency exploratory laparotomy. 

Intra-operative findings/description

Modified Makuuchi incision (J shape) was used for laparotomy, hemoperitoneum secondary to rupture of the right adrenal tumor was found, Hepatic flexure and transverse colon were mobilized, and Kocherisation of the duodenum was done. Right-sided retro peritoneum was explored, and careful dissection was done to isolate the right renal artery and vein. The right side of IVC (inferior vena cava) and the right renal vein were extensively stretched by the tumor. The tumor was carefully dissected by sharp dissection from the liver and the inferior vena cava. There was no obvious invasion of the tumor to segment six of the liver. The upper pole of the tumor was dissected free from the visceral surface of the liver, with ligation/clipping of vessels and the use of a ligature. The tumor was found to be ruptured at the inferior surface near the kidney. The lower border of the tumor was dissected from the upper pole of the kidney. Vessels feeding the adrenal tumor were ligated, and the tumor was removed and sent for histopathology. The tumor was 20 cm in the greatest dimension. It weighed around 960 grams with internal necrosis and hemorrhage. The total intraoperative blood loss was found to be 3.7 liters requiring a transfusion of 10 units of blood products. Homeostasis was secured, abdominal washout was done, and Redivac drains were placed in the retroperitoneal space. The patient was observed for a few days in the intensive care unit. She had an uneventful post-operative recovery and was discharged home on the 8th post-operative day.

Histopathology revealed High-Grade Adrenal Cortical Carcinoma. The tumor was confined to the adrenal cortex, 20 cm in the greatest dimension, and found to be ruptured with hemorrhage and necrosis. Margins were negative for carcinoma, and no lymphovascular invasion was identified. Histologically staged at pT2, N0, Mx. Immune staining showed a high Ki-67 index of 95 %. This histology was reported based on Weiss criteria. 

The case was discussed in the multi-disciplinary meeting, based on the clinical presentation, histology, and radiological imaging it was staged at T2N0M0, with preoperative tumor rupture and spillage and a recommendation was made for mitotane therapy as adjuvant treatment.

**Figure 7:**
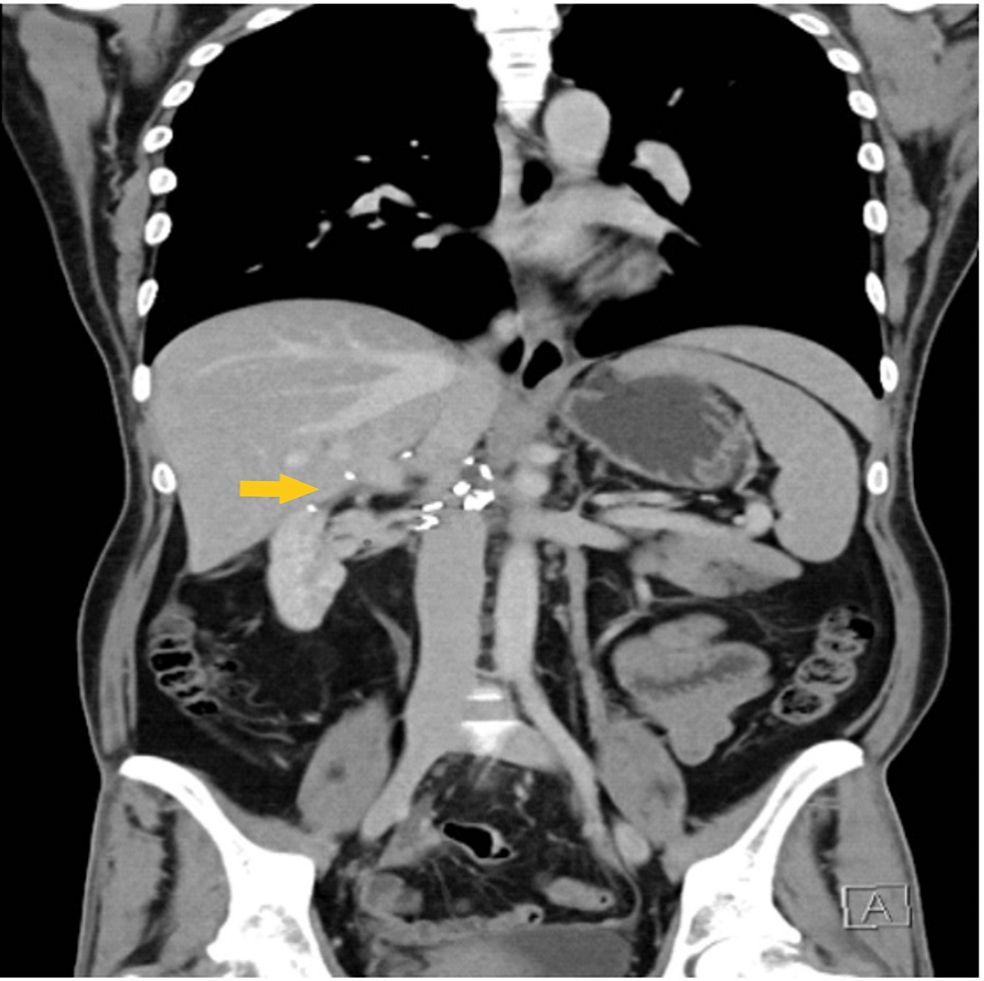
Post-operative CT scan (coronal view) at the one-month follow-up The image is showing clips at the surgical bed (yellow arrow) with no evidence of recurrence or metastases.

**Figure 8:**
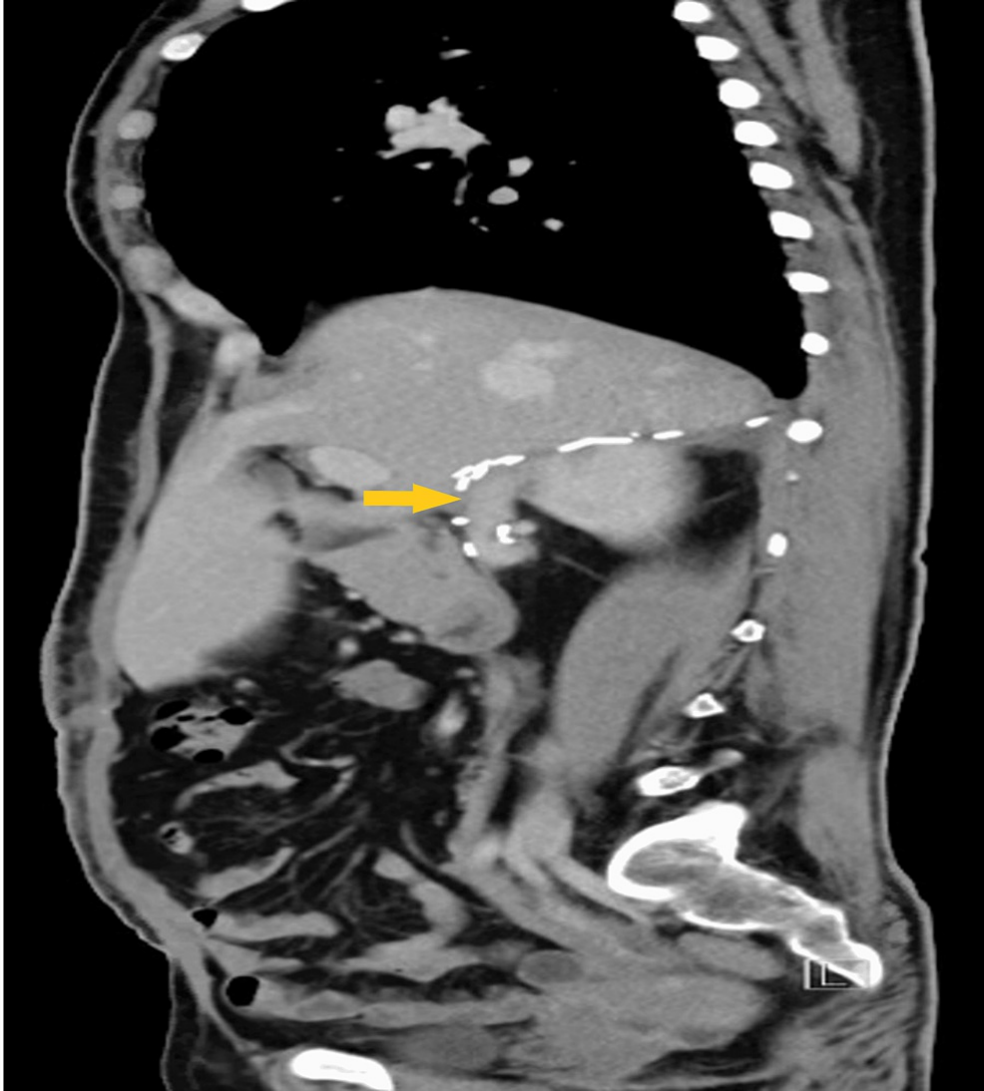
Post-operative CT scan (sagittal view) one month The follow-up image is showing clips at the surgical bed (yellow arrow) with no evidence of recurrence.

During her initial follow-up review in the clinic after 4 weeks, she was found to be asymptomatic and returned to normal activity as a housewife. Her follow-up surveillance CT scan (Figures 7, 8) performed before Mitotane therapy revealed a clean surgical bed with no signs of local recurrence or distant metastases except stable non-specific right upper lobe lung nodule from the initial pre-operative CT scan. A decision was made to proceed with monitoring lung nodules with the commencement of medical oncology treatment.

**Figure 9:**
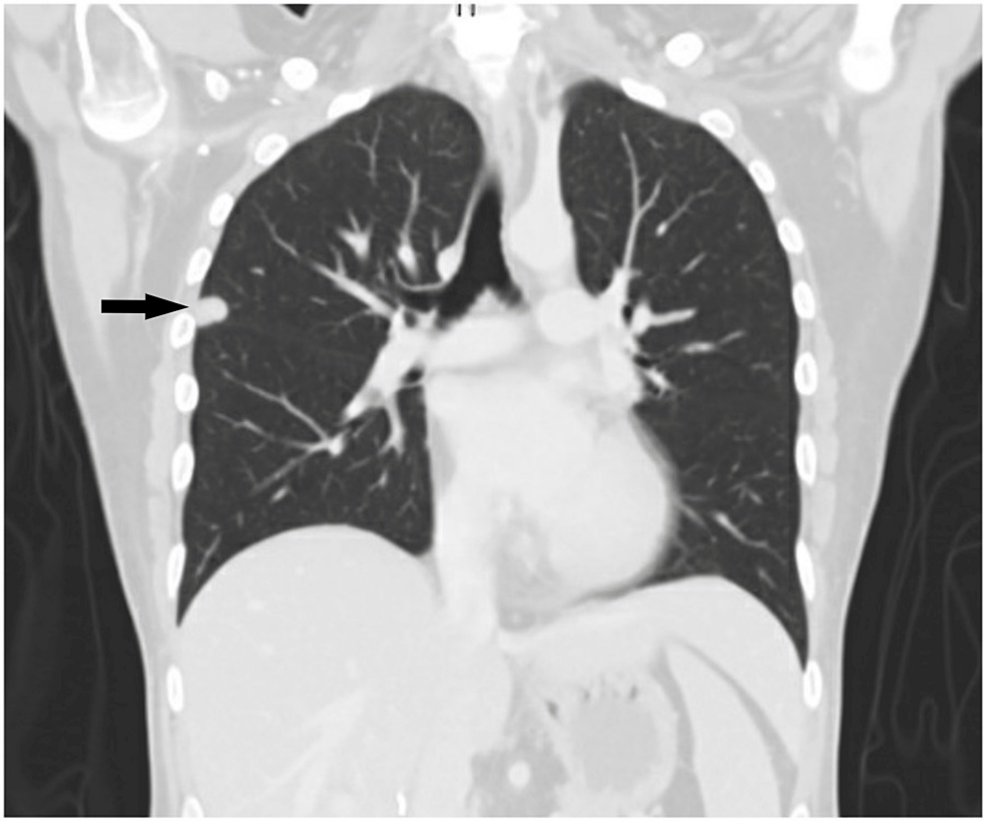
Post-operative CT scan (coronal view) on two-month follow-up The image is showing the progression of a previously noted lung nodule (black arrow).

**Figure 10:**
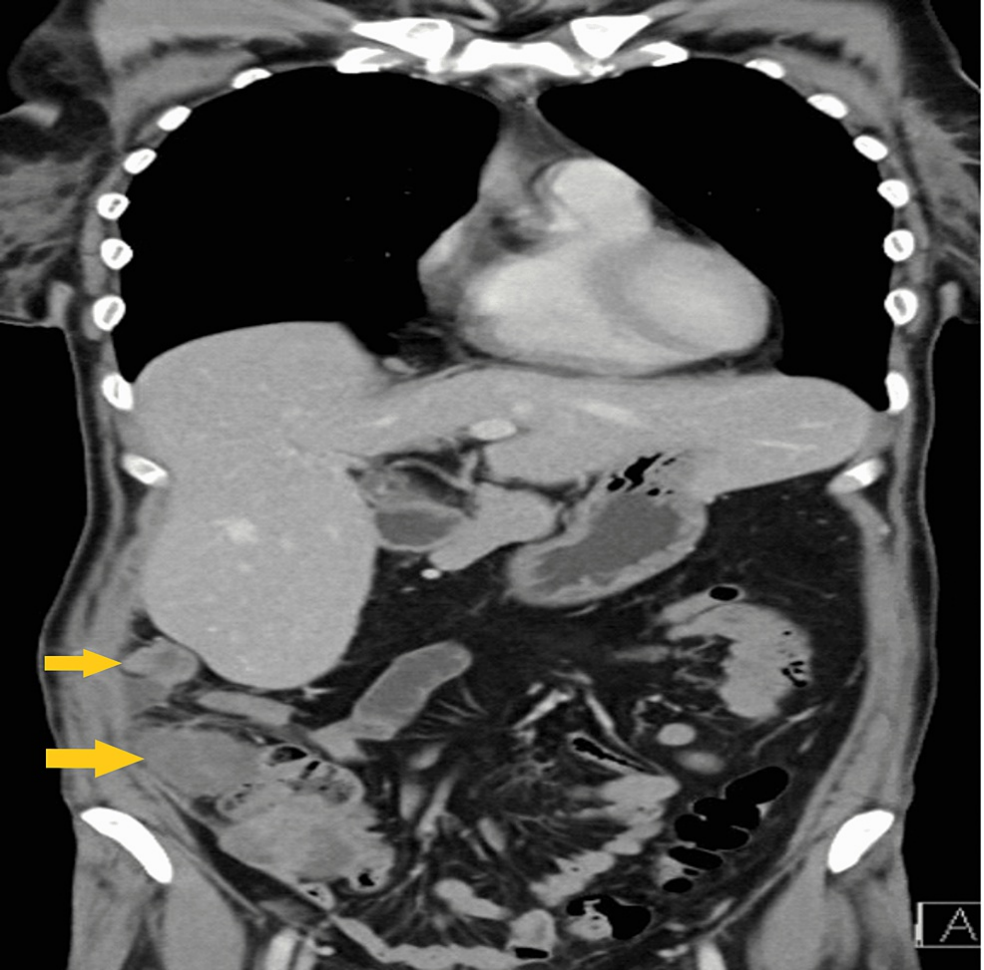
Post-operative CT scan (coronal view) on two-month follow-up The image is showing local intra-abdominal recurrence (yellow arrows).

In her subsequent clinic visit as part of the follow-up from surgery, the patient reported having vague lower abdominal pain with early satiety. The patient was compliant with mitotane therapy, she was under medical oncology and endocrinology follow-up also. Due to multiple high-risk factors for recurrence involved in her case, a CT scan was performed in 2nd month which revealed marked progression of right retroperitoneal, intra peritoneal metastatic disease and progression of solitary right pleural/lung nodule highly concerning for metastasis (Figures 9, 10).

The case was rediscussed in the multi-disciplinary meeting and a recommendation was made for starting second-line management like EDP-M (etoposide, doxorubicin, cisplatin-mitotane) as she had disease progression with mitotane therapy alone. Also considering her young age, the future possibility of further surgical options like redo surgery with debulking or hyperthermic intraperitoneal chemotherapy (HIPEC) surgery (currently under phase 2 trials) was suggested in case of expected response with second-line chemotherapy.

## Discussion

Adrenocortical cancer is a rare aggressive malignancy whose etiology remains unclear. Most of the time these tumors are sporadic, but they can be associated with other syndromes [8]. 50 to 60% of these tumors are functional with hypercortisolism being more common [3,7,8]. In our case, the tumor was non-functional. Approximately 40% of tumors have been reported to be non-functioning [3,7,8]. In the present case, non-functioning tumors may present with vague symptoms from abdominal mass, like nausea, vomiting, early satiety, abdominal fullness, or back pain. About 10 to 15% of the cases are diagnosed incidentally on imaging while evaluating non-tumor-related symptoms [4,8].

The prognosis of patients with adrenocortical cancer is poor with a median five-year survival rate of 16-35% [9]. Considering the poor prognosis, it is of paramount importance to critically evaluate adrenal masses for malignant potential. Endocrine evaluation not only helps in the diagnosis and perioperative preparation of the patient but also predicts the prognosis, as cortisol-secreting tumors have comparable worse prognoses [10].

Adrenal focus imaging like CT scans, MRI, and 18FDG-PET (18-fluorodeoxyglucose-positron emission tomography) scans are useful modalities for diagnosing ACC (adrenocortical carcinoma) [11,12]. High-risk features for malignancy on radiology include tumor size greater than 4 cm, Attenuation values above 10 HU ( Hounsfield unit) on CT scan, signal loss on chemical-shift imaging below 20 percent on MRI, high 18FDG-PET uptake, low central enhancement due to areas of hemorrhage and necrosis, and slow wash-out after intravenous injection of contrast material [8,11]. a staging CT scan should be done to rule out metastasis if malignancy is suspected. In our case, the CT scan prior to surgery revealed the following high-risk features suggestive of malignancy: large tumor size (13 cm), adrenal mass with attenuation >10 HU, heterogenous contrast enhancement, and central area of hypodensity. We did not have the chance to evaluate with MRI or PET scan preoperatively due to an emergency presentation.

These tumors should preferably be operated on by high-volume surgeons (15-20 adrenalectomies/year) [7,8]. Cases should be discussed at the multidisciplinary team meeting involving oncology surgeons, endocrinologists, pathologists, and medical oncologists.

Complete surgical resection (R0 status) is the cornerstone in the management of resectable adrenocortical cancers [7,8,13]. Tumor rupture or spillage and R2 resection are associated with high recurrence and poor overall survival [14]. An open surgical approach is the standard procedure in suspected or confirmed cases of adrenocortical cancer. The use of laparoscopic surgery in cancers smaller than 6 cm is debatable [7,8,13].

Large adrenal tumors may present with spontaneous rupture due to progressive stretch on the capsule by rapidly expanding tumors. Pheochromocytoma is the most common primary adrenal tumor showing spontaneous rupture followed by other tumors like myelolipomas, and secondary metastasis, while such incidence was least reported in the case of non-functional tumors [5]. In undiagnosed cases, spontaneous rupture may mimic acute abdomen with hemorrhagic shock leading to a diagnostic dilemma, as in our case.

Based on the severity of the hemorrhage and the clinical condition of the patient, management can vary from initial conservative management or angioembolization followed by planned surgery. Upfront aggressive surgery to control bleeding and resect the tumor may be required in case of failure of conservative strategy [5].

In our case, the patient had spontaneous rupture of the non-functional tumor, failure of attempted angioembolization, and the ongoing hemodynamic instability of the patient led to emergency laparotomy. She was operated on at a specialized center; we did achieve lifesaving control of ongoing bleeding as well as R0 resection of the tumor proven by negative margins on histopathology.

Evidence has been published on factors predicting the risk of recurrence based on tumor size, lymph node involvement, and the stage of disease (for example stage III & IV). Capsular invasion, cortisol secretion (in case of functioning tumor), Ki-67 index of >10% and tumor spillage during surgery [7,8]. 

Local and/or metastatic recurrence occurs in up to 50-70 percent of patients even after following R0 resection [15]. The time interval between resection and first recurrence varies between 1.5 to 150 months with a median of approximately one year [16]. Patients with early tumor recurrence 6-12 months have a poor prognosis [8]. Hence, close postoperative follow-up including clinical, hormonal, and imaging evaluation should be performed with CT scans and FDG-PET every three months for two years, and then every four to six months for more than five years [7,8]. Mitotane should be started in patients with a high risk of recurrence: stage III or R1 resection or Ki67 >10% as soon as possible (preferably within three months) [7,8]. Here the patient had unanticipated very early tumor recurrence at two months. In this case, the high-risk factors were young age, high tumor grade, large size, high proliferative index (Ki67 95%), and preoperative tumor rupture with hemorrhage and spillage of tumor cells.

## Conclusions

In our case, the patient unfortunately presented as a rare life-threatening hemorrhage secondary to spontaneous rupture of non-functioning adrenocortical cancer which was however managed appropriately in our territory care center. Despite adhering to the internationally accepted management guidelines, the tumor recurrence and metastasis were seen within two months of complete oncological resection. Our case did have high-risk factors such as large tumor size, tumor rupture with intra-peritoneal spillage, and a high Ki67 index.

Based on experience from this case, and a paucity of specialized centers dealing with this rare non-functioning adrenocortical cancer, we suggest a thorough workup with a multidisciplinary approach and reference to published standard guidelines in the management of these tumors; we would further suggest prioritizing early management, especially in dealing with large tumors, in order to prevent undue complications. Also, we would encourage clinicians to share and publish their experiences of this type of rare cancer to increase awareness and the development of innovative treatments.
